# Supplementary material for: Formation of twelve-fold iodine coordination at high pressure
Source: Nat Commun. 2022 Jan 20;13:412. doi: 10.1038/s41467-022-28083-4 (PMC8776873; doi:10.1038/s41467-022-28083-4)
Supplement: Supplementary file 1 — Supplementary Information [file 41467_2022_28083_MOESM1_ESM.pdf]

Supplementary Information

Formation of Twelve-fold Iodine Coordination at High Pressure

Yan Liu,[1] Rui Wang,[2] Zhigang Wang,[2] Da Li,[1*] and Tian Cui[1,3*]

[1]State Key Laboratory of Superhard Materials, College of Physics, Jilin University, Changchun 130012, P.R. China

[2]Institute of Atomic and Molecular Physics, Jilin University, Changchun 130012, P.R. China

[3]School of Physical Science and Technology, Ningbo University, Ningbo 315211, P.R. China

*Corresponding author. Email: dali@jlu.edu.cn (D.L.), cuitian@nbu.edu.cn (T.C.)

Table of Content

Supplementary Figures

Supplementary Figure 1 Crystal structure of $C2/m$-IN_3 at 80 GPa............................S4

Supplementary Figure 2 Electronic band structures of $C2/m$-IN_3 at 80 GPa.............S4

Supplementary Figure 3 Phonon dispersion curves of $R\bar{3}m$-IN_6...............................S5

Supplementary Figure 4 Schematic representation of IN_6 crystal structure...............S5

Supplementary Figure 5 Four-fold coordination of N in $R\bar{3}m$-IN_6............................S6

Supplementary Figure 6 Two-dimensional electron localization function (ELF) of $R\bar{3}m$-IN_6 at 100 GPa. S6

Supplementary Figure 7 Bond critical points in $R\bar{3}m$-IN_6...S7

Supplementary Figure 8 Electronic band structures of $R\bar{3}m$-IN_6 at 100 GPa............S7

Supplementary Figure 9 sp^3-hybridized N atom is notated by VSEPR.....................S8

Supplementary Figure 10 Projected density of states (PDOS) for hypothetical IN_0 and I_0N_6 at 100 GPa..S8

Supplementary Figure 11 Two-dimensional electron localization function (ELF) of $R\bar{3}m$-MN_6 (M = F, Cl, Br and I) at 100 GPa...S9

Supplementary Figure 12 Projected density of states (PDOS) for hypothetical MN_6 and MN_0 at 100 GPa..S10

Supplementary Figure 13 Crystal structure of $R\bar{3}m$-MN_6 (M = F, Cl, Br and I)......S11

Supplementary Tables

Supplementary Table 1 Structural information..S12

Supplementary Table 2 Molecular structure of IN_{12} fragment.................................S13

Supplementary Table 3 Component analysis of electron occupied MOs of IN_{12} S14

Supplementary Table 4 Basic physical properties of nitrogen and halogens.…........S17

Supplementary Table 5 ICOHP values in $R\bar{3}m$-MN_6 (M = F, Cl, Br and I)….........S18

Supplementary Figures

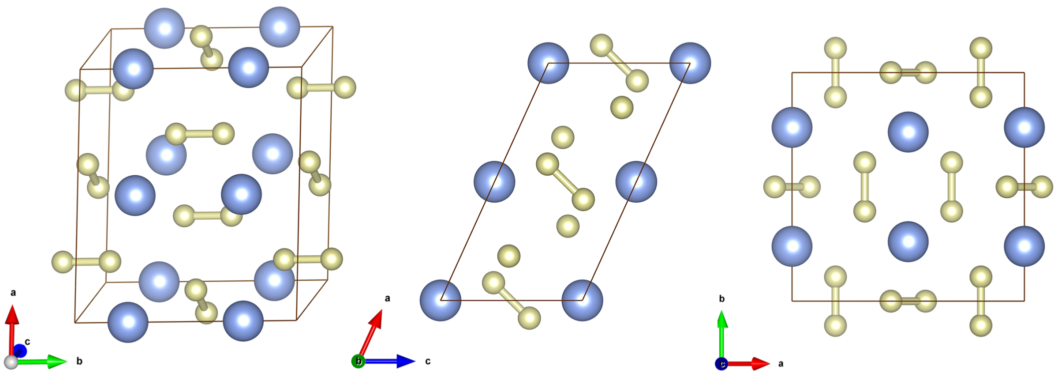

Supplementary Figure 1 Crystal structure of *C*2/*m*-IN_3 at 80 GPa. Two distinct diatomic N_2 lying on parallel and perpendicular to the *b*-axis, respectively.

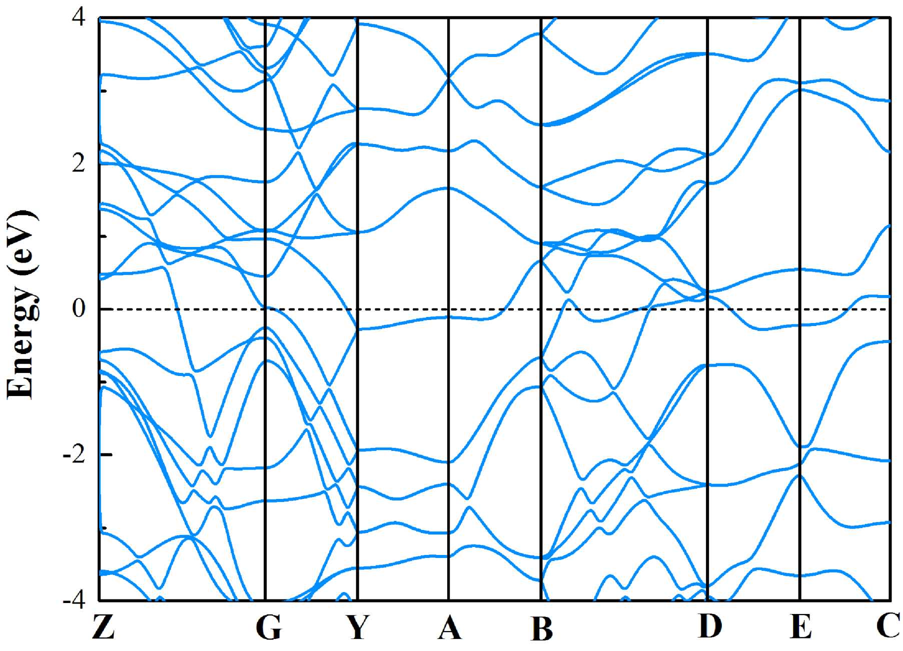

Supplementary Figure 2 Electronic band structures of *C*2/*m*-IN_3 at 80 GPa. The horizontal dashed line indicates the Fermi level.

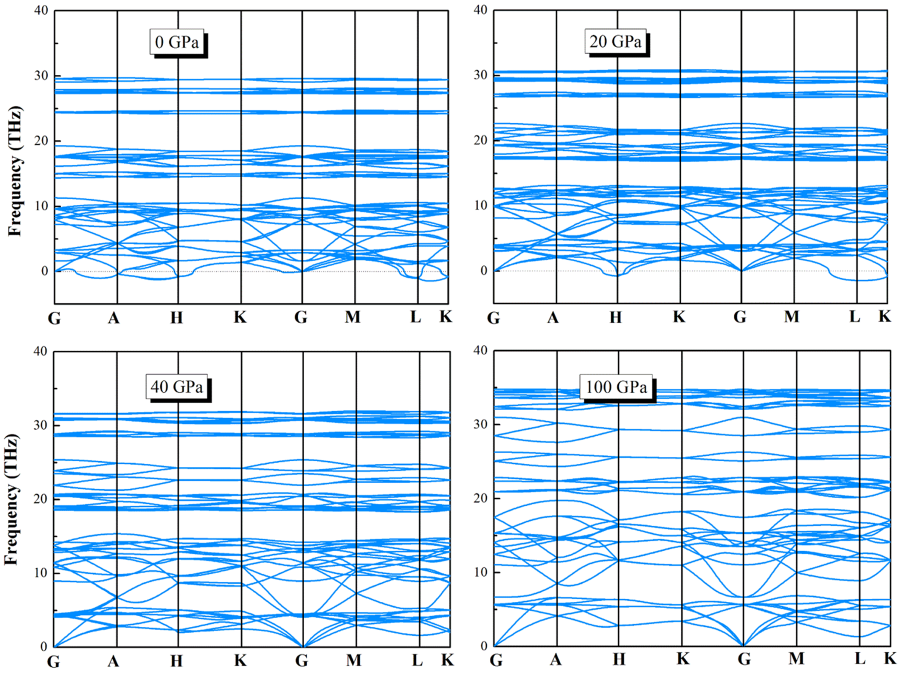

Supplementary Figure 3 Phonon dispersion curves of $R\bar{3}m$-IN_6.

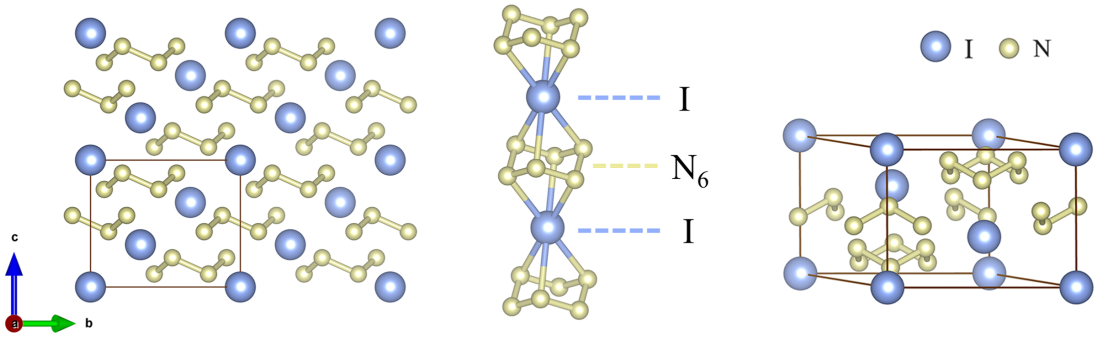

Supplementary Figure 4 Schematic representation of IN_6 crystal structure. I–N_6–I sandwiches structure in $R\bar{3}m$-IN_6 at 100 GPa. And the $R\bar{3}m$-IN_6 without showing I–N bonds, where chair-like N_6 are shown.

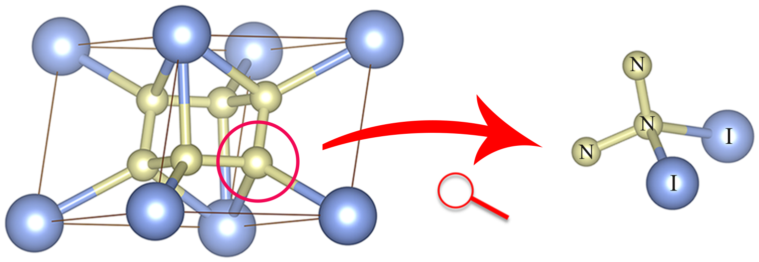

Supplementary Figure 5 The four-fold coordination of nitrogen, coordinated with two N and two I atoms in a primitive cell.

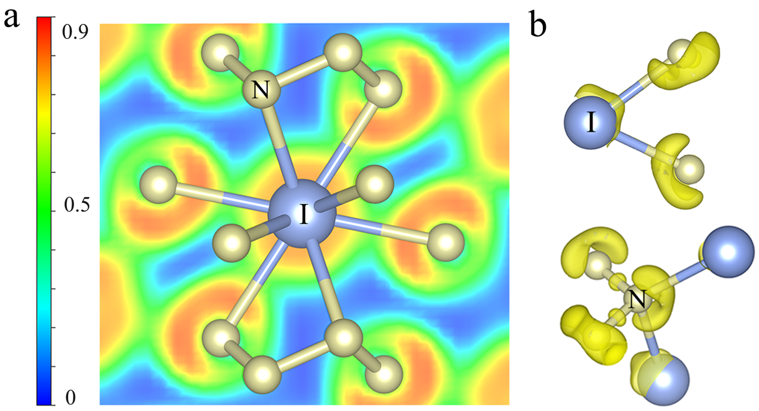

Supplementary Figure 6 Two-dimensional electron localization function (ELF) of $\boldsymbol{R\bar{3}m}$-$\mathbf{IN_6}$ at 100 GPa. **a** The ELF in a plane containing two kinds of I–N bonds. Red, green, and blue indicate perfect localization, an electron-gas-like state, and perfect delocalization, respectively. **b** ELF plots with an isosurface value of 0.83, showing covalent bonding around I and N atoms.

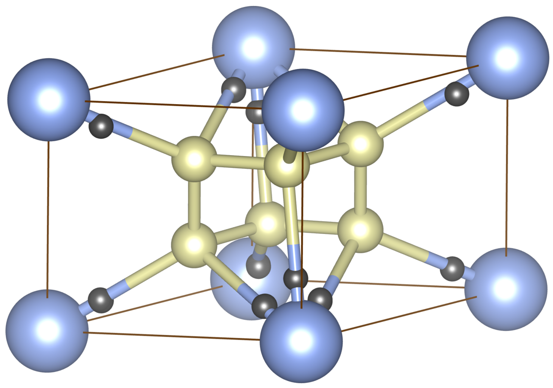

Supplementary Figure 7 Schematic representation of primitive cell of the $R\bar{3}m$-IN_6. The small black spheres correspond to the bond critical points (BCPs).

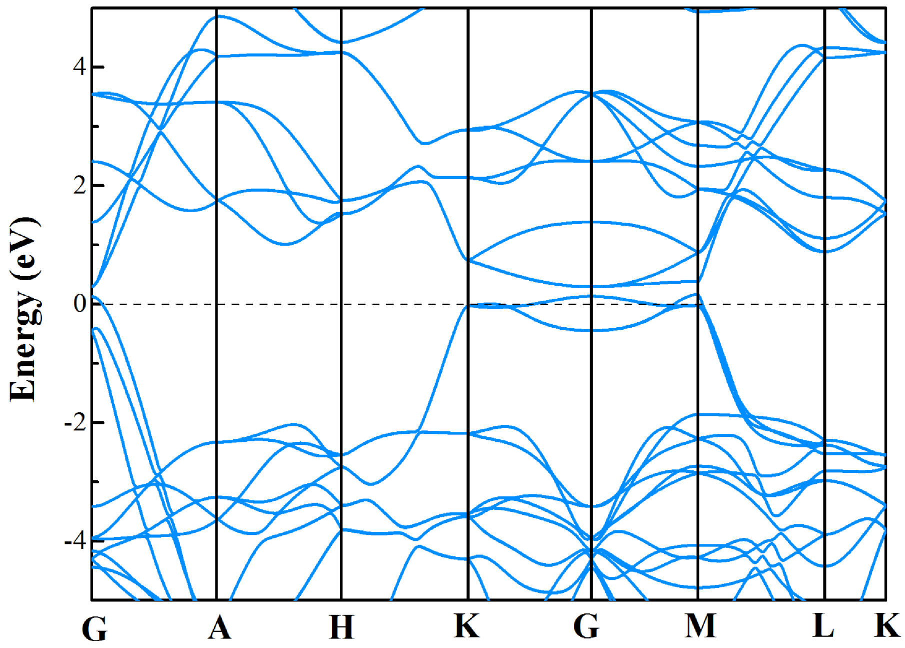

Supplementary Figure 8 Electronic band structures of $R\bar{3}m$-IN_6 at 100 GPa. The horizontal dashed line indicates the Fermi level.

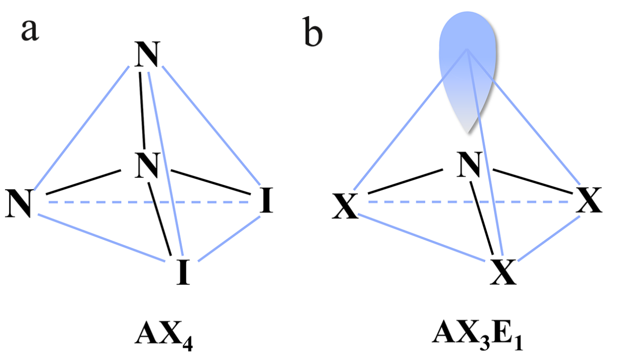

Supplementary Figure 9 The four-fold (**a**) and three-fold (**b**) coordination of N atoms (*sp*^{3}) are given in VSEPR notation. A and X represent the central atom (N) and neighbouring coordinated atoms, respectively, E represents the lone pair electrons.

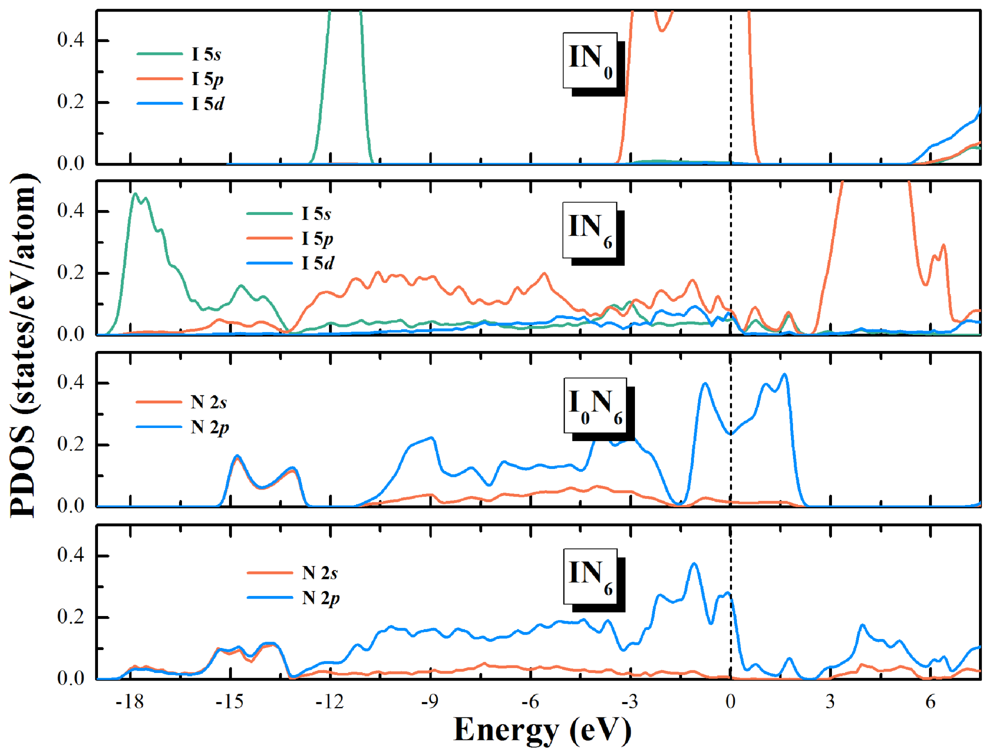

Supplementary Figure 10 Projected density of states (PDOS) for hypothetical IN_0 and I_0N_6 at 100 GPa. The dashed line indicates the Fermi energy.

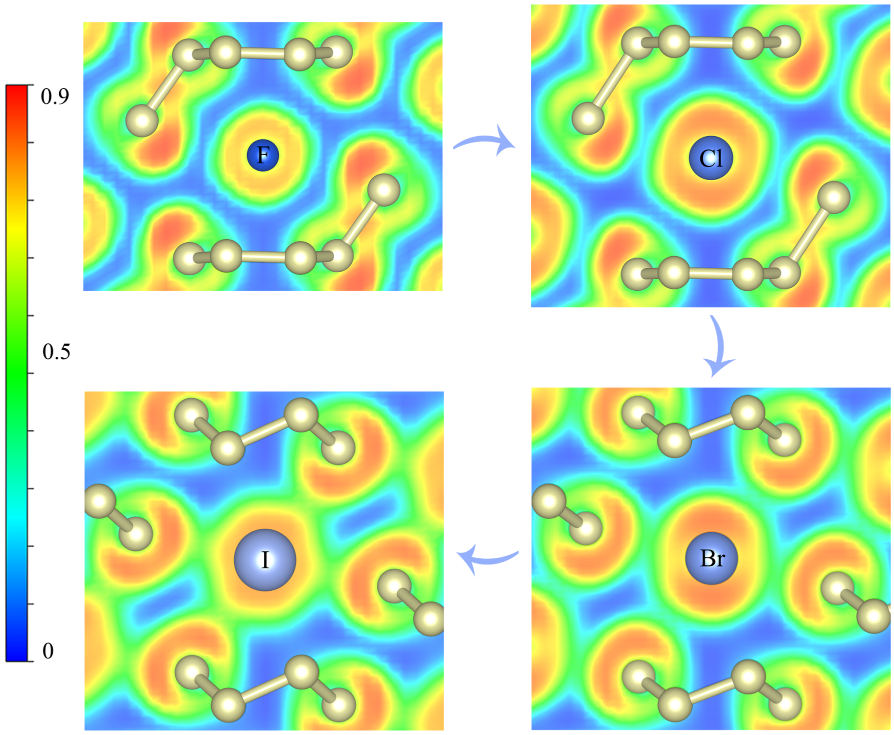

Supplementary Figure 11 The two-dimensional electron localization function (ELF) of $R\overline{3}m$-MN_6 (M = F, Cl, Br and I) compounds at 100 GPa. Red, green and blue corresponding to perfect localization, electron-gas-like state, and perfect delocalization, respectively.

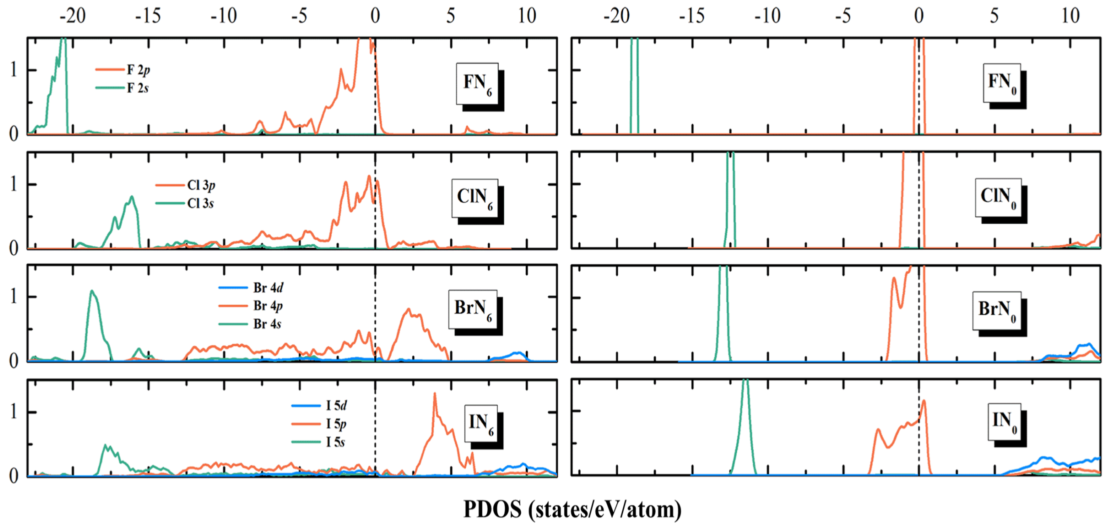

Supplementary Figure 12 Projected density of states (PDOS) for $R\bar{3}m$-MN_6 and MN_0 (M = F, Cl, Br and I) at 100 GPa. The dashed line indicates the Fermi energy.

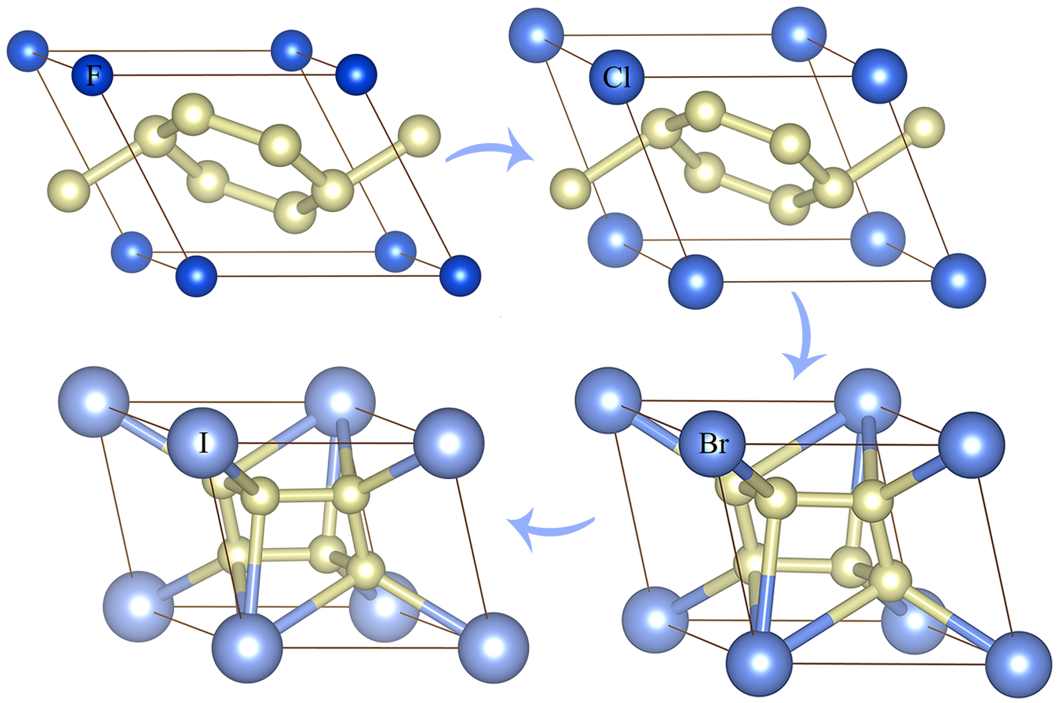

Supplementary Figure 13 The primitive cell of $R\bar{3}m$-MN_6 (M = F, Cl, Br and I) at 100 GPa.

Supplementary Tables

Supplementary Table 1 Unit-Cell parameters and atomic positions of the $C2/m$-IN_3 and $R\bar{3}m$-IN_6 at 80 and 100 GPa, respectively.

Structures	Lattice parameters (Å)	Structure parameters	Sites
	a = 6.65		
	b = 5.95	N1 (0.313,0.606,0.660)	8j
	c = 3.63	N2 (0.575,0.000,0.317)	4i
$C2/m$-IN_3	α = 90		
	β = 65.52	I (0.500,0.740,0.000)	4g
	γ = 90		
	a = 5.95		
	b = 5.95	N (0.908,0.454,0.769)	18h
	c = 4.35		
$R\bar{3}m$-IN_6	α = 90.00		
	β = 90.00	I (0.667,0.333,0.333)	3a
	γ = 120.00		

Supplementary Table 2 Molecular structure of IN_{12} fragment with D_{3d} point group symmetry.

13<br># .xyz file	x	y	z
**N**	7.0690	2.4130	7.9080
**N**	5.9730	4.3100	7.0140
**N**	7.5430	3.2340	5.5640
**N**	9.4090	4.3110	5.5640
**N**	7.5430	5.3890	5.5640
**N**	9.2610	2.4140	7.0160
**N**	7.0690	6.2080	7.9060
**N**	10.3570	4.3120	7.9080
**N**	9.2610	6.2090	7.0140
**N**	8.7870	3.2340	9.3580
**N**	8.7870	5.3890	9.3580
**N**	6.9210	4.3110	9.3580
**I**	8.1650	4.3110	7.4610

Supplementary Table 3 The component analysis of electron occupied molecular orbitals (MOs) of IN_{12} in Fig. 4 of main text. Due to D_{3d} point group symmetry of the structure, twelve N atoms have two positions and they are divided into two compositions. The composition of six N atoms, whose position is closer to the I atom, is called 6N1 (six N1 atoms). The other composition is called 6N2 (six N2 atoms). The E represents the energy of MOs and the unit is eV. The OS and os represent the orbital symmetry of IN_{12} and compositions (I, 6N1, 6N2), respectively. The c is composition of IN_{12}.

Spin up						Spin down					
MOs	E (eV)	OS	percentage	os	c	MOs	E (eV)	OS	percentage	os	c
HOMO	Not selected					HOMO-1	-7.30	A1.g	38.57%	A1.g	6N1
						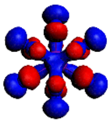			37.82%	A1.g	6N1
									22.64%	A1.g	6N2
									1.57%	5d	I
									1.11%	5s	I
HOMO-2	-7.49	E1.g:2	39.45%	E1.g:2	6N1	HOMO-2	-7.39	E1.g:2	45.15%	E1.g:2	6N2
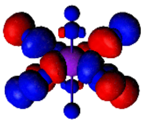			24.67%	E1.g:2	6N2	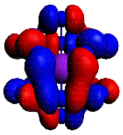			26.76%	E1.g:2	6N1
			16.50%	E1.g:2	6N2				12.99%	E1.g:2	6N2
			13.58%	E1.g:2	6N1				6.24%	E1.g:2	6N1
			3.39%	E1.g:2	6N1				5.34%	E1.g:2	6N2
			1.56%	E1.g:2	6N2				3.95%	E1.g:2	6N1
			1.07%	5d	I						
HOMO-3	-7.49	E1.g:1	39.45%	E1.g:1	6N1	HOMO-3	-7.39	E1.g:1	45.15%	E1.g:1	6N2
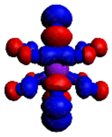			24.67%	E1.g:1	6N2	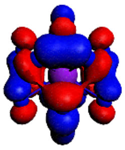			26.76%	E1.g:1	6N1
			16.50%	E1.g:1	6N2				12.99%	E1.g:1	6N2
			13.58%	E1.g:1	6N1				6.24%	E1.g:1	6N1
			3.39%	E1.g:1	6N1				5.34%	E1.g:1	6N2
			1.56%	E1.g:1	6N2				3.95%	E1.g:1	6N1
			1.07%	5d	I						
HOMO-4	-7.89	E1.g:2	46.57%	E1.g:2	6N1	HOMO-8	-9.79	E1.g:2	46.27%	E1.g:2	6N1
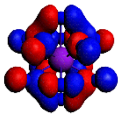			22.82%	E1.g:2	6N2	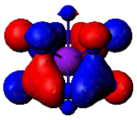			42.40%	E1.g:2	6N2
			13.23%	E1.g:2	6N2				4.51%	E1.g:2	6N2
			9.98%	E1.g:2	6N2				1.77%	E1.g:2	6N1
			4.01%	E1.g:2	6N1				1.72%	E1.g:2	6N2
			3.06%	E1.g:2	6N1				1.05%	5d	I
HOMO-5	-7.89	E1.g:1	46.57%	E1.g:1	6N1	HOMO-9	-9.79	E1.g:1	46.27%	E1.g:1	6N1
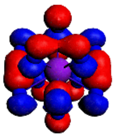			22.82%	E1.g:1	6N2	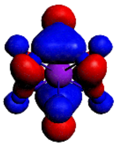			42.40%	E1.g:1	6N2
			13.23%	E1.g:1	6N2				4.51%	E1.g:1	6N2
			9.98%	E1.g:1	6N2				1.77%	E1.g:1	6N1
			4.01%	E1.g:1	6N1				1.72%	E1.g:1	6N2
			3.06%	E1.g:1	6N1				1.05%	5d	I

HOMO-9	-9.53	A1.u	86.14%	A1.u	6N1	HOMO-11	-10.17	E1.g:2	79.31%	E1.g:2	6N1
			13.49%	A1.u	6N2				10.13%	E1.g:2	6N2
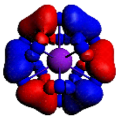						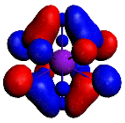			2.88%	E1.g:2	6N2
									2.06%	E1.g:2	6N1
									1.85%	E1.g:2	6N2
									1.26%	5d	I
HOMO-10	-9.84	E1.g:2	34.01%	E1.g:2	6N1	HOMO-12	-10.17	E1.g:1	79.31%	E1.g:1	6N1
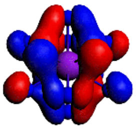			32.89%	E1.g:2	6N2	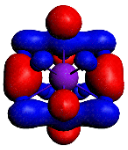			10.13%	E1.g:1	6N2
			21.12%	E1.g:2	6N2				2.88%	E1.g:1	6N2
			5.63%	E1.g:2	6N1				2.06%	E1.g:1	6N1
			1.91%	E1.g:2	6N1				1.85%	E1.g:1	6N2
			1.33%	5d	I				1.26%	5d	I
			1.30%	E1.g:2	6N2						
HOMO-11	-9.84	E1.g:1	34.01%	E1.g:1	6N1	HOMO-15	-10.30	A1.u	94.38%	A1.u	6N1
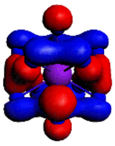			32.89%	E1.g:1	6N2	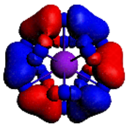			5.40%	A1.u	6N2
			21.12%	E1.g:1	6N2						
			5.63%	E1.g:1	6N1						
			1.91%	E1.g:1	6N1						
			1.33%	5d	I						
			1.30%	E1.g:1	6N2						
HOMO-18	-12.46	E1.u:2	28.39%	5p	I	HOMO-17	-12.50	A2.u	33.81%	5p	I
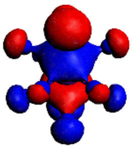			22.09%	E1.u:2	6N1	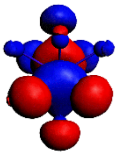			26.53%	A2.u	6N2
			16.01%	E1.u:2	6N1				16.66%	A2.u	6N2
			12.86%	E1.u:2	6N2				10.55%	A2.u	6N2
			7.49%	6p	I				7.94%	6p	I
			6.84%	E1.u:2	6N1				3.07%	A2.u	6N$_1$
			3.98%	E1.u:2	6N2				1.73%	A2.u	6N1
			1.99%	E1.u:2	6N2				1.21%	A2.u	6N1
			1.74%	E1.u:2	6N2						
HOMO-19	-12.46	E1.u:1	28.39%	5p	I	HOMO-18	-12.54	E1.u:2	34.81%	E1.u:2	6N1
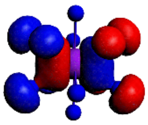			22.09%	E1.u:1	6N1	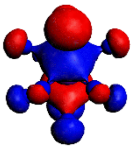			30.57%	5p	I
			16.01%	E1.u:1	6N1				11.37%	E1.u:2	6N1
			12.86%	E1.u:1	6N2				7.02%	6p	I
			7.49%	6p	I				6.05%	E1.u:2	6N2
			6.84%	E1.u:1	6N1				5.62%	E1.u:2	6N2
			3.98%	E1.u:1	6N2				4.04%	E1.u:2	6N1
			1.99%	E1.u:1	6N2						
			1.74%	E1.u:1	6N2						
HOMO-20	-13.48	A2.u	34.40%	A2.u	6N2	HOMO-19	-12.54	E1.u:1	34.81%	E1.u:1	6N1
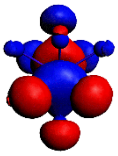			27.56%	5p	I	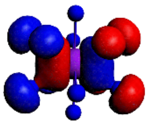			30.57%	5p	I
			15.34%	A2.u	6N2				11.37%	E1.u:1	6N1
			12.86%	A2.u	6N2				7.02%	6p	I

			6.26%	6p	I				6.05%	E1.u:1	6N2
			2.37%	A2.u	6N1				5.62%	E1.u:1	6N2
			1.56%	A2.u	6N1				4.04%	E1.u:1	6N1
			1.01%	A2.u	6N1						
HOMO-21	-17.73	A1.g	55.86%	A1.g	6N1	HOMO-22	-18.08	A1.g	31.36%	5s	I
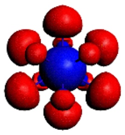			24.88%	5s	I	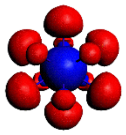			30.88%	A1.g	6N1
			6.25%	A1.g	6N2				24.25%	A1.g	6N2
			5.40%	6s	I				5.84%	6s	I
			3.27%	A1.g	6N2				4.75%	A1.g	6N1
			3.14%	A1.g	6N1				1.61%	A1.g	6N2
			1.63%	A1.g	6N2				1.38%	A1.g	6N2
HOMO-33	-26.58	A1.g	46.72%	5s	I	HOMO-32	-26.41	A1.g	49.60%	5s	I
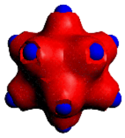			30.49%	A1.g	6N2	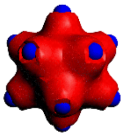			24.78%	A1.g	6N1
			17.25%	A1.g	6N1				20.77%	A1.g	6N2
			4.62%	A2.g	6N2				4.14%	A2.g	6N1
			3.69%	A1.g	6N1				3.64%	A1.g	6N2

Here, the molecular orbitals in list are characterized by a composition of more than 30% of 6N1 composition or I atoms and a large contribution of the *p* orbitals of 6N1. Although there are the other MOs with a large proportion of 6N1, they are made up more of *s* orbital or vacant orbital of N atoms than *p* orbitals.

Supplementary Table 4 Some physical properties of nitrogen and halogens.

**Element**	Valence shell electron configurations	Covalent radii (pm)	Electronegativity (Pauling)	Ionization energy ($kJ\cdot mol^{-1}$)
N	$2s^22p^3$	70	3.04	1402
F	$2s^22p^5$	64	3.98	1681
Cl	$3s^23p^5$	99	3.16	1251
Br	$4s^24p^5$	114	2.96	1140
I	$5s^25p^5$	133	2.66	1008

S: Ionization energies cited in this table are from C. E. Moore, *Ionization Potentials and Ionization Limits Derived from the Analyses of Optical Spectra*, National Standard Reference Data Series, U.S. National Bureau of Standards, NSRDS-NBS 34, Washington, DC, 1970.

Supplementary Table 5 Selected N−N, M−N, and M−M bond lengths and average ICOHP values in the structure of $R\bar{3}m$-MN_6 (M = F, Cl, Br and I) at 100 GPa.

Structures	Bond	d / Å	average ICOHP / eV / bond
	N–N	1.31	–14.89
**FN_6**	F–N	2.25	–0.09
	F–F	3.66	0.00
	N–N	1.31	–14.81
**ClN_6**	Cl–N	2.45	–0.18
	Cl–Cl	3.76	0.01
	N–N	1.32	–14.51
**BrN_6**	Br–N	2.35	–0.98
	Br–Br	3.70	–0.02
	N–N	1.36	–12.75
**IN_6**	I–N	2.27	–2.04
	I–I	3.73	–0.03
